# Retraction Note: The LMCD1-AS1/miR-526b-3p/OSBPL5 axis promotes cell proliferation, migration and invasion in non-small cell lung cancer

**DOI:** 10.1186/s12890-023-02596-0

**Published:** 2023-08-11

**Authors:** Rui Hu, Yankai Yu, Haining Wang

**Affiliations:** Department of Thoracic Surgery, Shengli Oilfield Central Hospital, 31 Jinan Road, Dongying, 257034 Shandong China

**Retraction Note: BMC Pulmonary Medicine (2022) 22:30**

10.1186/s12890-022-01820-7

The Editor has retracted this article. After publication, the authors contacted the journal requesting a correction to Figs. 2E and F and 5D and E, and S3D due to the use of incorrect images. Further checks by the Editor found that the study used the SPC-A1 cell line as a model of lung cancer, but it has been reported to be contaminated with HeLa cells, which makes it unsuitable for lung cancer research.

The Editor therefore no longer has confidence in the presented data and the conclusions of this study.

Haining Wang has agreed to this retraction but not the wording of this retraction notice. Rui Hu and Yankai Yu have not responded to any correspondence from the editor or publisher about this retraction.

